# Clinical relevance of potentially inappropriate medications and potential prescribing omissions according to explicit criteria—a validation study

**DOI:** 10.1007/s00228-022-03337-8

**Published:** 2022-06-01

**Authors:** Naldy Parodi López, Staffan A. Svensson, Susanna M. Wallerstedt

**Affiliations:** 1 Department of Pharmacology, Sahlgrenska Academy, University of Gothenburg, Box 431, 405 30 Gothenburg, Sweden; 2 Närhälsan Kungshöjd Health Centre, Gothenburg, Sweden; 3 Närhälsan Hjällbo Health Centre, Gothenburg, Sweden; 4 HTA-Centrum, Sahlgrenska University Hospital, Gothenburg, Sweden

**Keywords:** Clinical relevance, EU(7)-PIM list, Older people, Potentially inappropriate medications, Potential prescribing omissions, Screening Tool of Older Persons’ Prescriptions (STOPP), Screening Tool to Alert to Right Treatment (START), Swedish set of criteria

## Abstract

**Purpose:**

To investigate the clinical relevance of potentially inappropriate medications (PIMs) and potential prescribing omissions (PPOs), and to evaluate the association between PIMs/PPOs and inadequate drug treatment.

**Methods:**

PIMs/PPOs, concordantly identified by two physicians applying the STOPP/START criteria, the EU(7)-PIM list, and a Swedish set in 302 consecutive older primary care patients, were assessed regarding clinical relevance for the specific patient. The physicians determined, in consensus, whether an action related to the medication was medically justified prior to the next regular consultation. If so, the drug treatment was categorised as inadequate, and if not, the treatment was considered adequate.

**Results:**

In all, 259 (86%) patients had 1010 PIMs/PPOs, 150 (15%) of which, in 81 (27%) patients, were assessed as clinically relevant (kappa: 0.26). A total of 75 (50%) clinically relevant PIMs and PPOs were prioritised for medical action before the next regular consultation. Action-requiring clinically relevant PIMs most often concerned acetylsalicylic acid (ASA) for primary prevention (four out of 68 patients on ASA). The corresponding PPOs concerned beta-blockers in ischaemic heart disease (four out of 61 patients with this condition). When an overall medical perspective was applied, 164 (63%) out of 259 patients with PIMs/PPOs were assessed as having adequate treatment. In adjusted logistic regression, number of PIMs and/or PPOs and number of drugs were associated with inadequate drug treatment.

**Conclusion:**

One in seven PIMs/PPOs may be clinically relevant, half of these not of priority for medical action. Cautious interpretation is warranted when PIMs/PPOs are used as outcome measures.

## Introduction

Criteria to identify potentially inappropriate prescribing, i.e. potentially inappropriate medications (PIMs) or potential prescribing omissions (PPO), are often used to describe the adequacy of drug treatment in older people [1, 2]. The presence of PIMs/PPOs has been linked to hospital readmissions and high health care costs [3, 4], and they have also been suggested for inclusion in core outcome sets for the evaluation of effects of interventions to improve prescribing practices [5–7].

Over the last decades, several sets of criteria have been developed to identify PIMs and PPOs in older people, mostly using a consensus (Delphi) methodology and including the EU(7)-PIM list [8], the Screening Tool of Older Persons’ Prescriptions (STOPP), and the Screening Tool to Alert to Right Treatment (START) [9]. In Sweden, a set of criteria, developed by the Swedish National Board of Health and Welfare, has been in use since 2004 [10].

Research regarding the clinical relevance of identified PIMs/PPOs is sparse [11, 12]. Further validation of the clinical relevance of criteria that form the basis for identification of PIMs/PPOs is therefore essential, preferably including an overall assessment of the patient’s medical situation rather than merely assessing isolated drugs or drug-diagnosis combinations. For this purpose, a medical approach, taking into account the health status of the specific patient as well as the clinical context in which medical prioritisations have to be made, could contribute insights.

The aim of this study was to investigate the clinical relevance of PIMs and PPOs identified in older primary care patients using three established sets of criteria. We also wanted to evaluate the association between the presence of PIMs/PPOs and inadequate drug treatment, when assessed from an overall medical perspective.

## Methods

In this validation study, we used a cohort of patients included in a previous cross-sectional study investigating the association between recorded medication reviews and adequacy of drug treatment management in 302 consecutive patients ≥ 65 years of age who had attended a physician’s consultation in either of two primary health care centres in Sweden [13]. The patients had a median age of 74 years (range: 65–99), and 178 (59%) were female. In the previous study, two physicians, both specialists (in family medicine: N.P.L., S.A.S.; and in clinical pharmacology: S.A.S.), independently identified PIMs/PPOs according to three sets of criteria [8–10, 14] and assessed the clinical relevance of each identified PIM/PPO. They then decided on the adequacy of drug treatment for each patient from an *overall* medical perspective, first independently and then in consensus discussions, taking into account that medical prioritisations have to be made in clinical practice. The drug treatment was categorised as *adequate* if no action related to the medication was considered medically justified at the individual level, prior to the next regular consultation. Conversely, the drug treatment was categorised as *inadequate* if this premise was not fulfilled, i.e. if a switch or the withdrawal of a drug, the ordering of a laboratory test, the retrieval of more information about the patient, or arranging an extra consultation was considered medically justified. All assessments were based on printed copies of the electronic medical records over the 2½ years preceding the physician consultation, including laboratory tests, hospital discharge records, vaccinations, prescriptions, and interaction alerts integrated into the medical record system [15].

**Fig. 1:**
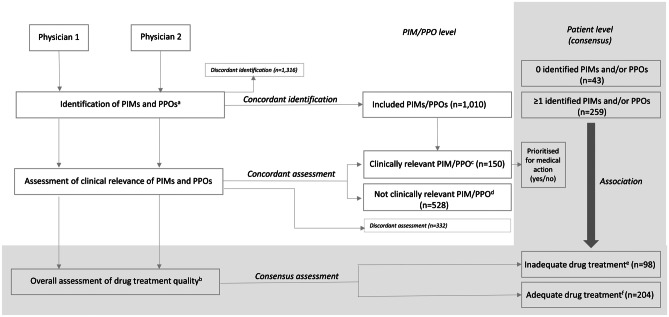
Drug treatment assessments**.** Abbreviations: *PIM* potentially inappropriate medication, *PPO* potential prescribing omission. ^a^Three sets of criteria: the European Union (EU)(7)-PIM list, the Screening Tool of Older Persons’ Prescriptions (STOPP)/Screening Tool to Alert to Right Treatment (START), and the Swedish set of criteria developed by the National Board of Health and Welfare. ^b^From a medical perspective, taking into account the health condition of the specific patient and medical priorities that have to be made in primary health care. ^c^PIMs/PPOs assessed by both physicians as either clinically relevant, or of uncertain clinical relevance but with a related medical action suggested. ^d^PIMs/PPOs assessed by both physicians as either not clinically relevant, or of uncertain clinical relevance, with no related medical action suggested. ^e^Defined as one or more actions related to the medication being considered medically justified at the individual level, prior to the next regular consultation, according to two physicians in consensus, e.g. a switch or the withdrawal of a drug, ordering of a laboratory test, retrieval of more information about the patient, or arranging an extra visit. ^f^Defined as no action related to the medication being considered medically justified at the individual level, prior to the next regular consultation, according to two physicians in consensus

In the present study, data regarding identified PIMs/PPOs and their clinical relevance were recorded (Fig. 1). In all, 452 PIM and 53 PPO criteria, partly overlapping, were applied: the EU(7)-PIM list, 282 PIMs [8]; the STOPP/START criteria version 2, 80 PIMs and 34 PPOs [9]; and the Swedish set of criteria developed by the National Board of Health and Welfare, 87 PIMs and 22 PPOs [10, 14]. PIMs/PPOs concordantly identified by both assessors were included in the analysis. The clinical relevance of each PIM/PPO, from the perspective of a primary care physician, was assessed and categorised as either (i) clinically relevant; (ii) of uncertain clinical relevance, but with one or more related medical actions suggested; (iii) not clinically relevant; or (iv) of uncertain clinical relevance, with no related medical action suggested. The PIMs/PPOs that were concordantly assessed by both physicians as belonging to category (i) or (ii) were collapsed into a *Clinically relevant* category, while those concordantly assessed as (iii) or (iv) were collapsed into a *Not clinically relevant* category. For clinically relevant PIMs/PPOs, the physicians performed consensus discussions to determine if a related action was medically prioritised at the individual level. The PIMs/PPOs with discordant assessments regarding clinical relevance are reported separately.

### Statistics

Statistical analyses were conducted using SPSS Statistics for Windows, version 26.0 (IBM Corp., Armonk, NY, USA). Descriptive statistics are presented as numbers (percentages) and/or median (interquartile range or range). To compare categorical data, the chi-square test was performed, and for discrete data, the Mann–Whitney *U* test was conducted. Kappa statistics were used to evaluate the inter-rater agreement between the two physicians regarding their assessments of the clinical relevance of PIMs/PPOs. We also calculated the kappa value without the START criterion concerning influenza vaccination. Logistic regression was performed to obtain crude and adjusted odds ratios, including 95% confidence intervals (CIs), for the association between the number of PIMs/PPOs and inadequately managed drug treatment. Variables included in the models were age, sex (female versus male), multi-dose drug dispensing (yes versus no), and number of regular drugs, reflecting the burden of disease [16]. Multicollinearity was evaluated by examining tolerance levels.

## Results

The patients were treated with a median of five regular drugs (range: 0–17). Characteristics of patients with adequate and inadequate drug treatment are presented in Table 1. Drugs frequently listed on the medication list included paracetamol (*n* = 127; 42%), omeprazole (*n* = 72; 24%), acetylsalicylic acid (ASA) (*n* = 68; 23%), atorvastatin (*n* = 68; 23%), and metoprolol (*n* = 61; 20%).

**Table 1:** Patient characteristics, and adequacy of drug treatment

		Inadequate^a^ (*n* = 98)	Adequate^b^ (*n* = 204)	*P*-value
Age, years		74 (69–82)	74 (69–81)	0.57
Female sex		56 (57)	122 (60)	0.66
Nursing home resident		14 (14)	17 (8)	0.11
Multi-dose drug dispensing		14 (14)	19 (9)	0.19
Drug treatment	Number of regular drugs	6 (4–9)	4 (2–7)	< 0.001
	≥ 5 regular drugs	68 (69)	85 (42)	< 0.001
Common conditions	Hypertension	69 (70)	134 (66)	0.41
	eGFR < 60 mL/min	42 (43)	60 (29)	0.021
	Osteoarthritis	31 (32)	60 (29)	0.69
	Type 2 diabetes	37 (38)	48 (16)	0.010
	Insomnia	25 (26)	53 (26)	0.93
	Chronic ischaemic heart disease	27 (28)	34 (17)	0.027
	Depression	24 (24)	31 (15)	0.050
	Obstipation	18 (18)	36 (18)	0.88
	Urinary incontinence	26 (27)	27 (13)	0.004
	Atrial fibrillation	22 (22)	25 (12)	0.022
	Dyspepsia	23 (23)	19 (9)	0.001

Data are presented as numbers (percentages) or median (interquartile range)

*eGFR* estimated glomerular filtration rate

^a^Defined as one or more actions related to the medication being considered medically justified at the individual level, prior to the next regular consultation, according to two physicians in consensus, e.g. a switch or the withdrawal of a drug, ordering of a laboratory test, retrieval of more information about the patient, or arranging an extra visit

^b^Defined as no action related to the medication being considered medically justified at the individual level, prior to the next regular consultation, according to two physicians in consensus

A total of 746 PIMs and 264 PPOs were concordantly identified in 259 (86%) patients and consequently included in the analysis (Fig. 1): a median of one PIM (range: 0–17) and one PPO per patient (range: 0–7). For 488 (65%) PIMs and 190 (72%) PPOs, both assessors made the same decision regarding clinical relevance for the specific patient (kappa: 0.23 and 0.37, respectively), and 100 (13%) PIMs and 50 (19%) PPOs were categorised as clinically relevant with somewhat varying proportions between the sets of criteria (Table 2). A total of 75 (50%) out of 150 clinically relevant PIMs/PPOs were prioritised for medical action before the next regular consultation according to consensus assessment by both physicians.

**Table 2:** Clinical relevance of identified PIMs and PPOs, taking into account the health condition of the specific patient

		Total	Clinically relevant^a^	Not clinically relevant^b^	Discordant assessment^c^	Inter-rater agreement (kappa)^d^
PIMs/PPOs	All	1010	150 (15)	528 (52)	332 (33)	0.26
PIMs	All	746	100 (13)	388 (52)	258 (35)	0.23
	EU(7)-PIM list	277	21 (8)	179 (65)	77 (28)	0.21
	STOPP	136	13 (10)	77 (57)	46 (34)	0.16
	Swedish set	333	66 (20)	132 (40)	135 (41)	0.21
PPOs	All	264	50 (19)	140 (53)	74 (28)	0.37
	START	205	36 (18)	121 (59)	48 (23)	0.44
	Swedish set	59	14 (24)	19 (32)	26 (44)	0.16

Data are presented as numbers (percentages)

*EU* European Union, *PIMs* potentially inappropriate medications, *PPOs* potential prescribing omissions, *START* Screening Tool to Alert to Right Treatment, *STOPP* Screening Tool of Older Persons’ Prescriptions, *Swedish set* Swedish set of criteria developed by the National Board of Health and Welfare

^a^PIMs/PPOs assessed by both physicians as either (i) clinically relevant or (ii) of uncertain clinical relevance, but with a related medical action suggested

^b^PIMs/PPOs assessed by both physicians as either (iii) not clinically relevant or (iv) of uncertain clinical relevance, but with no related medical action suggested

^c^PIMs/PPOs discordantly assessed by the two physicians, regarding the clinical relevance for the specific patient

^d^Between the assessors’ categorisation of PIMs and/or PPOs, concordantly identified, as being clinically relevant or not

At the patient level, one or more clinically relevant PIMs/PPOs were identified in 81 (27%) patients (range: 0–6 PIMs/PPOs per patient). Analysed separately, one or more clinically relevant PIMs and PPOs were found in 50 patients (17%; range: 0–6 PIMs per patient) and 39 patients (13%; range: 0–3 PPOs per patient), respectively.

Potentially inappropriate medications frequently identified included proton pump inhibitors (PPIs) (220 PIMs in 76 (25%) patients), drugs for insomnia (116 PIMs in 58 (19%) patients), and benzodiazepines (62 PIMs in 21 (7%) patients) (Table 3). The PIMs most frequently assessed as clinically relevant concerned inappropriate use of ASA for primary prevention of cardiovascular disease (*n* = 8; 12% of all patients on ASA), followed by propiomazine against insomnia (*n* = 7; 12% of patients with drugs against insomnia), and use of furosemide with no clinical indication (*n* = 7; 12% of all patients on furosemide). An action prior to the next regular visit was considered prioritised, given the medical context, in four (6%) out of 68 patients on ASA, three (5%) out of 61 patients taking drugs against insomnia, and three (5%) out of 57 patients on loop diuretics (all furosemide).

**Table 3:** PIMs most often identified using three sets of indicators of prescribing quality (≥ 5%) or most often being assessed as clinically relevant (*n* > 5)

	Total *n* (% of all)	Identified using the:			≥ 1 clinically relevant PIM^a,b^	A prioritised medical action suggested before the next regular visit^b^
		EU(7)-PIM list	STOPP criteria	Swedish set		
PPIs > 8 weeks or without an evidence-based clinical indication	76 (25)	73	7	60	5 (7)	1 (1)
Hypnotic Z-drugs or zopiclone > 3.75 mg/d or zolpidem > 5 mg/d or other drugs for insomnia including propiomazine but not benzodiazepines	58 (19)	47	44	12	7 (12)	3 (5)
Presence of benzodiazepines or use of benzodiazepines > 4 weeks or use of a long-acting benzodiazepine, e.g. diazepam	21 (7)	9	21	9	1 (5)	0 (0)
Weak opioids, e.g. codeine or codeine > 2 weeks or tramadol	17 (6)	14	0	17	4 (24)	3 (18)
COX inhibitors > 2 weeks or naproxen > 500 mg/d or naproxen > 1 week or use of diclofenac or etoricoxib	14 (5)	14	0	4	6 (43)	3 (21)
Loop diuretic without a clinical indication	14 (5)	0	7	12	7 (50)	3 (21)
ASA for primary prevention of cardiovascular disease	10 (3)	0	0	10	8 (80)	4 (40)

Data are presented as numbers (percentages)

*ASA* acetylsalicylic acid, *COX* cyclooxygenase, *EU* European Union, *PIMs* potentially inappropriate medications, *PPI* proton pump inhibitor, *STOPP* Screening Tool of Older Persons’ Prescriptions, *Swedish set* Swedish set of criteria developed by the National Board of Health and Welfare

^a^PIM assessed by both physicians as either (i) clinically relevant or (ii) of uncertain clinical relevance, but with a related medical action suggested

^b^Percentage of patients from each PIM subcategory

The most frequently identified potentially prescribing omission was the START criterion related to vaccination against influenza, which was found in 172 (57%) patients, assessed as clinically relevant in 27 (16%) patients, and prioritised for action prior to the next regular consultation in one patient. The second most common PPO concerned the lack of a beta-blocker in patients with chronic ischaemic heart disease; five out of 16 identified omissions, in 61 patients suffering from the condition, were considered clinically relevant and in four of these cases, action was prioritised before the next regular visit (Table 4).

**Table 4:** PPOs most often identified using two sets of indicators of prescribing quality (≥ 3%) or most often being assessed as clinically relevant (*n* > 5)

	Total *n* (% of all)	Identified using the:		≥ 1 clinically relevant PPO^a,b^	A prioritised medical action suggested before the next regular visit^b^
		START criteria	Swedish set		
Seasonal trivalent influenza vaccine annually	172 (57)	172	0	27 (16)	1 (1)
Beta-blocker for chronic ischaemic heart disease	16 (5)	4	16	5 (31)	4 (25)
Statin for chronic ischaemic heart disease	14 (5)	4	12	4 (29)	0 (0)
ACE inhibitor or ARB and/or dihydropyridine calcium channel blocker and/or thiazide diuretic for hypertension	12 (4)	0	12	3 (25)	2 (17)
Pneumococcal vaccine at least once after 65 years of age	9 (3)	9	0	3 (33)	1 (11)

Data are presented as numbers (percentages)

*ACE* angiotensin-converting enzyme, *ARB* angiotensin II receptor blocker, *PPOs* potential prescribing omissions, *START* Screening Tool to Alert to Right Treatment, *Swedish set* Swedish set of criteria developed by the National Board of Health and Welfare

^a^PPO assessed by both physicians as either (i) clinically relevant or (ii) of uncertain clinical relevance, but with a related medical action suggested

^b^Percentage of patients from each PPO subcategory

Overall, considering each patient’s state of health in the clinical context of primary care, 164 (63%) out of 259 patients with one or more PIMs/PPOs were found to have adequate drug treatment. Among those with one or more clinically relevant PIMs/PPOs, 31 (38%) patients had adequate drug treatment.

Logistic regression revealed that the number of PIMs and/or PPOs, as well as the number of regular drugs, was associated with inadequately managed drug treatment, with overlapping 95% CIs (Table 5). In a post hoc regression analysis excluding the PIM/PPO variable, given that similar odds ratios were obtained for the variable “number of regular drugs”, the adjusted odds for inadequate drug treatment increased by the number of drugs: 1.27 (95% CI 1.16–1.39). The tolerance level was > 0.6 for all variables included in the models, indicating that multicollinearity was not a problem.

**Table 5:** Factors associated with inadequate drug treatment management. Confidence intervals not crossing the line of unity, showing a statistically significant difference between the groups, are in bold type

	Crude OR (95% CI)	Adjusted OR^b^ (95% CI)		
		Model 1	Model 2	Model 3
Number of PIMs/PPOs	**1.31 (1.19–1.44)**	**1.29 (1.16–1.43)**		
Number of PIMs	**1.30 (1.18–1.43)**		**1.25 (1.13–1.40)**	
Number of PPOs	**1.56 (1.20–2.02)**			**1.50 (1.14–1.98)**
Age, years	1.02 (0.98–1.05)	1.00 (0.96–1.04)	1.00 (0.97–1.04)	0.99 (0.95–1.03)
Sex (female versus male)	0.90 (0.55–1.46)	0.77 (0.44–1.32)	0.75 (0.43–1.28)	0.92 (0.54–1.56)
Multi-dose drug dispensing (yes versus no)	1.62 (0.78–3.39)	**0.35 (0.13–0.93)**	0.45 (0.18–1.14)	0.54 (0.22–1.35)
Number of regular drugs^a^	**1.25 (1.15–1.36)**	**1.19 (1.08–1.32)**	**1.20 (1.09–1.33)**	**1.27 (1.16–1.39)**

*CI* confidence interval, *OR* odds ratio

^a^Number of drugs from 0 until 10, and ≥ 11

^b^For each adjusted model, all variables presented with figures were included

## Discussion

In this study, we show that one out of seven identified PIMs/PPOs, using three established sets of criteria for potentially inappropriate prescribing, was clinically relevant for the specific individual, and that half of these were not prioritised for medical action. Furthermore, almost two-thirds of the patients with one or more PIMs/PPOs had adequate drug treatment when an overall medical perspective was applied. The odds for some aspect of the drug therapy being considered inadequate increased similarly for both the number of regular drugs and the number of identified PIMs and/or PPOs.

One in eight identified PIMs, and one in five identified PPOs, was considered clinically relevant. Furthermore, every other clinically relevant PIM/PPO was not prioritised for a related medical action prior to the next regular visit. These findings indicate that the three sets of criteria for identifying inappropriate drug use in older people detect many potential problems that, on closer scrutiny, turn out to be of fairly low relevance. The sets of criteria therefore appear to be sensitive rather than specific, at least when applied in primary care. The present findings are even more conspicuous than those of a study where the first version of the STOPP/START criteria was applied, in older patients hospitalised for hip fracture, where half of the PIMs/PPOs were assessed to be clinically relevant [11]. The varying degrees of clinical relevance may be explained by the fact that the assessments of PIMs/PPOs in our study applied an overall medical perspective. In addition, fewer PIMs/PPOs may be clinically relevant in a younger and probably healthier population in primary care. This interpretation is supported by the overall assessment of the drug treatments in our study; few PIMs/PPOs that were considered clinically relevant necessitated taking prioritised action before the next regular visit.

The PIMs most often considered clinically relevant were ASA for primary prevention, drugs against insomnia, and a loop diuretic with no indication. These findings echo those of a previous study on hospitalised patients [11], and may reflect the complexity of withdrawing these drugs once they have been initiated. Also, the benefit-risk balance for ASA in primary prevention has been scientifically controversial [17], and physicians as well as patients may be reluctant to discontinue this regime, once established. Evidence gaps like this one may, at least in part, explain the minimal inter-rater agreement between the physicians regarding the clinical relevance of the PIMs/PPOs.

Interestingly, the most frequent PIM, that is, being treated with a PPI for more than 8 weeks or without an evidence-based clinical indication, was clinically relevant in less than one-tenth of the cases. When medical prioritisations in primary health care were taken into account, another four-fifths did not merit a related medical action. These findings may contribute insights to the interpretation of previous findings that long-term use of PPI without a clear reason is prevalent in older people [18].

The PPO most often considered clinically relevant was the absence of vaccination against seasonal influenza. In only one patient out of 27, however, was this deemed of sufficient priority to merit action before the next regular visit. Prioritisation is a central task in the medical assessment of individual patients, and in primary care, there are often several, possibly conflicting, issues at hand during a medical consultation [13, 19]. Nevertheless, our results regarding the clinical relevance of the influenza vaccine criterion, combined with varying kappa values with and without this criterion, suggest that physicians consider the influenza vaccination important, but that other health problems may be more urgent to attend to in the clinical context.

As mentioned previously, the inter-rater agreement concerning the clinical relevance of identified PIMs and PPOs was low in our study. Furthermore, since kappa regarding clinical relevance was calculated for PIMs/PPOs concordantly identified by both assessors, leaving out discordantly identified PIMs/PPOs, the inter-rater agreement can be expected to be overestimated. This finding is noteworthy as the assessors had a similar clinical background and assessed patients from a clinical context they were both familiar with. Furthermore, although a global assessment was made, it is reasonable to assume that the three sets of criteria would influence and to some degree harmonise their assessments. The low concordance between assessors with relevant expertise, despite these aspects, may illustrate the complexity of evaluating medical treatments and prioritisations in primary care. In fact, reliability may be an issue also in seemingly less complex pharmacotherapeutic assessments; a substantial proportion of the PIMs/PPOs were identified by one assessor but not the other. These results diverge from the initial reliability studies of the STOPP/START criteria [20, 21], and will be further elucidated in a separate study.

Our findings that most identified PIMs and PPOs were not considered clinically relevant, and that even fewer merited some medical action prior to the next regular visit, suggest limitations regarding their validity to give a true reflection of the quality of drug treatment. These results are consistent with validity issues previously reported for drug-specific indicators of prescribing quality [22]. In addition, another study found that most drug-related problems identified during medication reviews were not associated with the STOPP/START criteria [23]. Interestingly, medication reviews based on these tools have recently failed to show patient-relevant effects in a large-scale European trial [24], further supporting that validity may be an issue. Taken together, it may be questionable to include PIMs/PPOs in core outcome sets for improved prescribing practices, as has been suggested [5–7].

Given these caveats regarding validity, it is pertinent to ask whether there may be simpler proxies for inadequate drug treatment than PIMs and PPOs. Interestingly, our regression analyses showed that the association between PIMs/PPOs and inadequate drug treatment was similar to that between the number of regularly used drugs and such treatment. One may speculate that the number of PIMs/PPOs and the number of drugs both mirror the complexity of care. In other settings, the number of drugs has proved quite a good proxy for the burden of disease [16], and increasing morbidity is naturally linked to more challenging prescribing decisions. Interestingly, one of our regression models showed that multi-dose drug dispensing was associated with lower odds for inadequate drug treatment. However, in general, the literature points in the opposite direction; prescribing practices have been shown to be problematic among patients with multi-dose drug dispensing [25–27]. Nonetheless, previous studies have not taken into account the overall medical perspective in assessing the adequacy of prescribing. Clearly, these topics merit further investigation.

### Strengths and limitations

An important strength of the present study is that it provides insights into the clinical relevance of three established sets of criteria: the EU(7)-PIM list, the STOPP/START criteria version 2, and the Swedish set of criteria developed by the National Board of Health and Welfare. Such evidence has hitherto been lacking. The assessment procedure, where two specialist physicians with in-depth knowledge and experience of working in primary care first independently and then jointly examined all cases, increases the likelihood of capturing all relevant problems related to pharmacotherapy. Indeed, assessments of the quality of drug treatment by two specialist physicians have previously been shown to yield reliable results [28]. Furthermore, the results may have an acceptable generalisability as consecutive primary care patients were included; a large variety of patients present in this setting.

Noteworthy limitations include the extent of discordant identifications of PIMs/PPOs, as well as the minimal agreement between two experienced physicians’ assessments regarding the clinical relevance of concordantly identified PIMs/PPOs. These observations deserve further attention in future research. Furthermore, it should be noted that the Swedish criteria set is primarily intended for those 75 years of age and older, whereas both the STOPP/START criteria and the EU(7)-PIM list were fully applicable to the study cohort. Nevertheless, our results suggest that the Swedish set of criteria may not be less relevant. Another limitation may be that we obtained information from the patients’ medical records only, and that there may have been relevant facts that were not documented in these records, for instance verbal information from a patient that they were in fact not currently using one of the drugs on the list. Still, the assessors had access to most of the information any primary care physician would normally have.

## Conclusion

This study shows that six in seven identified PIMs/PPOs in older patients, detected by three sets of criteria, may not be clinically relevant. Furthermore, every second clinically relevant PIM/PPO was not deemed to be of sufficient priority to merit some medical action prior to the patient’s next regular visit to the primary health care centre. Our results imply that caution is warranted when interpreting previous research using PIMs and PPOs as outcome measures.

## Data Availability

The database from this study is not publicly available owing to Swedish data protection laws. The data can be shared with authorised persons after they have applied and obtained approval from the Swedish Ethical Review Authority.
